# Supplementary material for: The Impact of Cancer-Related Fatigue on HRQOL in Survivors of Childhood Cancer: A DCCSS LATER Study
Source: Cancers (Basel). 2022 Jun 9;14(12):2851. doi: 10.3390/cancers14122851 (PMC9221067; doi:10.3390/cancers14122851)
Supplement: Supplementary file 1 [file cancers-14-02851-s001.zip › cancers-1731940-supplementary.pdf]

Supplementary material

Supplementary Table S1. Health-related quality of life concepts measured by the SF-36 and TAAQOL.

Dimension	Abbreviation	Definition	Number of items
**SF-36**			
Physical functioning	PF	Limitations in physical activities because of health problems	10
Social functioning	SF	Limitations in social activities because of physical or emotional problems	2
Bodily pain	BP	Experience of physical pain in daily life	2
General mental health	MH	Psychological distress and well-being	5
Role limitations (physical)	RP	Limitations in usual role/work-related activities because of physical health problems	4
Role limitations (emotional)	RE	Limitations in usual role/work-related activities because of emotional problems	3
Vitality	VT	Feelings of energy and fatigue	4
General health perceptions	GH	Perception of current and future health including resistance to illness	5
**TAAQOL**			
Sleep	SL	Problems/limitations concerning sleeping (lying awake, sleeping restlessly)	4
Sexuality	SE	Problems/limitations concerning sex (frequency, satisfaction)	2
Cognitive functioning	CO	Problems/limitations concerning cognitive functioning (concentrating, remembering)	4

Supplementary Table S2. Confounder variables included in multivariable analysis and their categories.

Variable of interest	Questionnaire item (when applicable)	Categories
Age at assessment	Date of assessment – date of birth	In years continuous
BMI	Length (cm) and weight (kg) measured during clinic visit	Underweight: BMI <18.5<br>Healthy weight: BMI between 18.5 and 25<br>Overweight: BMI between 25 and 30<br>Obesity: BMI ≥30
Employment status	Do you currently have work? Yes/no	Employed: Currently employed<br>Unemployed: Currently unemployed
Educational level	What is the highest level of education you have completed? Answer options: primary education, vocational education, preparatory secondary vocational education, secondary vocational education, school of higher general secondary education, pre-university education, higher vocational education, university, special school.	Low: Primary education, vocational education, special school<br>Middle: Preparatory secondary vocational education, secondary vocational education, school of higher general secondary education, pre-university education<br>High: Higher vocational education, university
Sleep quality	Seven PSQI component scores and total scores were calculated using scoring instructions described elsewhere [40]	Good sleeper: PSQI total score ≤5<br>Poor sleeper: PSQI total score >5
Anxiety	The outcomes of the seven items of the HADS anxiety subscale were added up and the total score was used to indicate person's as having anxiety yes/no.	No anxiety: HADS anxiety subscale score <8<br>Anxiety: HADS anxiety subscale score ≥8
Depression	The outcomes of the seven items of the HADS depression subscale were added up and the total score was used to indicate person's as having depression yes/no.	No depression: HADS depression subscale score <8<br>Depression: HADS depression subscale score ≥8
Number of somatic comorbidities	In 2013 a questionnaire about health issues was completed and used to indicate whether a participant suffered one or more health issues as classified by Streefkerk et al. [41]	0: Zero comorbidities<br>1-2: One or two comorbidities<br>>2: More than two comorbidities

Abbreviations: BMI=Body Mass Index; PSQI=Pittsburg Sleep Quality Index; HADS=Hospital Anxiety and Depression Scale

Supplementary Table S3. Overview missing values

Variable	Number of missing values (%)
SF-36	
PF	40 (2.4)
RP	41 (2.4)
RE	49 (2.9)
BP	45 (2.7)
MH	53 (3.1)
VT	53 (3.1)
GH	53 (3.1)
SF	44 (2.6)
TAAQOL	
SL	12 (0.7)
SE	60 (3.5)
CO	10 (0.6)
PSQI poor sleeper (yes/no)	53 (3.1)
HADS anxiety (yes/no)	79 (4.7)
HADS depression (yes/no)	81 (4.8)
BMI	48 (2.8)
Employment status	18 (1.1)
Educational level	17 (1.0)
Number of somatic comorbidities	328 (19.4)

Abbreviations: PF=Physical Functioning, RP=Role Physical, RE=Role Emotional, BP=Bodily Pain, MH=Mental Health, VT=Vitality, GH=General Health, SL=Sleep, SE=Sexuality, CO=Cognitive Functioning. The variables sex, age at assessment, primary childhood diagnosis, treatment, and chronic fatigue were not included in the table as no missing values were present.

Supplementary Table S4. Comparison participants vs. non-participants.

Characteristic	Participants (n=1695) N (%)	Non-participants (n=2296)* N (%)	p-value [e]	ES [f]
**Female sex**	817 (48.2)	937 (40.8)	<0.001	0.07
**Decade of birth**			0.230	0.04
<1960	20 (1.2)	22 (0.9)		
1960-1969	143 (8.4)	160 (7.0)		
1970-1979	436 (25.7)	582 (25.3)		
1980-1989	654 (38.6)	875 (38.1)		
≥1990	442 (26.1)	658 (28.6)		
**Age at diagnosis**			0.167	0.04
0-5	770 (45.4)	1093 (47.6)		
5-10	458 (27.0)	625 (27.2)		
10-15	370 (21.8)	437 (19.0)		
15-18	97 (5.7)	141 (6.1)		
**Primary childhood cancer diagnosis** [a]			0.526	0.05
Leukemia	581 (34.3)	781 (34.0)		
Non-Hodgkin lymphoma [b]	210 (12.4)	261 (11.4)		
Hodgkin lymphoma	121 (7.1)	162 (7.1)		
CNS	158 (9.3)	263 (11.5)		
Neuroblastoma	97 (5.7)	122 (5.3)		
Retinoblastoma	8 (0.5)	16 (0.7)		
Renal tumors	193 (11.4)	251 (10.9)		
Hepatic tumors	17 (1.0)	25 (1.1)		
Bone tumors	101 (6.0)	120 (5.2)		
Soft tissue tumors	124 (7.3)	169 (7.4)		
Germ cell tumors	56 (3.3)	95 (4.1)		
Other and unspecified [c]	29 (1.7)	31 (1.4)		
**Childhood cancer treatment** [d]			<0.001	0.12
Surgery only	109 (6.4)	266 (11.6)		
Chemotherapy, no radiotherapy	917 (54.1)	1293 (56.3)		
Radiotherapy, no chemotherapy	93 (5.5)	132 (5.7)		
Radiotherapy and chemotherapy	568 (33.5)	575 (25.0)		
No treatment/treatment unknown	8 (0.5)	30 (1.3)		
**Recurrence**			0.103	0.03
No	1468 (86.6)	2028 (88.3)		
Yes	227 (13.4)	268 (11.7)		

**Non-participants were invited to participate but did not return or complete the fatigue and HRQOL questionnaires*

[a] *Diagnostic groups included all malignancies covered by the third edition of the International Classification of Childhood Cancer (ICCC-3) as well as multifocal Langerhans cell histiocytosis.*

[b] *Includes all morphology codes specified in the ICCC-3 under lymphomas and reticuloendothelial neoplasms, except for Hodgkin lymphomas. Also includes multifocal Langerhans cell histiocytosis.*

[c] *Includes all morphology codes specified in the ICC-3 under other malignant epithelial neoplasms and malignant melanomas and other and unspecified malignant neoplasms.*

[d] *Treatment data included primary treatment and all recurrences.*

[e] *Chi-Square test*

[f] *Effect size, calculated as Cramér's V (<0.1=little, 0.1=low, 0.3=medium, 0.5=high).*

Complete names of the Dutch LATER Study Group

Birgitta Versluys, Martha Grootenhuis, Flora van Leeuwen, Lideke van der Steeg, Geert Janssens, Hanneke van Santen, Jaap den Hartogh, Lilian Batenburg, Hanneke de Ridder, Nynke Hollema, Lennart Teunissen, Anke Schellekens)
